# Neural crest precursors from the skin are the primary source of directly reprogrammed neurons

**DOI:** 10.1016/j.stemcr.2024.10.003

**Published:** 2024-10-31

**Authors:** Justin J. Belair-Hickey, Ahmed Fahmy, Wenbo Zhang, Rifat S. Sajid, Brenda L.K. Coles, Michael W. Salter, Derek van der Kooy

**Affiliations:** 1 Donnelly Centre, Department of Molecular Genetics, University of Toronto, Toronto, ON, Canada; 2 Program in Neurosciences and Mental Health, The Hospital for Sick Children, Toronto, ON, Canada; 3 Department of Laboratory Medicine and Pathobiology, University of Toronto, Toronto, ON, Canada; 4 Department of Physiology, University of Toronto, Toronto, ON, Canada

**Keywords:** neural crest, direct reprogramming, precursor cell differentiation, fate mapping, skin, neuron, germ layer, embryonic lineage, plasticity, cell identity

## Abstract

Direct reprogramming involves the conversion of differentiated cell types without returning to an earlier developmental state. Here, we explore how heterogeneity in developmental lineage and maturity of the starting cell population contributes to direct reprogramming using the conversion of murine fibroblasts into neurons. Our hypothesis is that a single lineage of cells contributes to most reprogramming and that a rare elite precursor with intrinsic bias is the source of reprogrammed neurons. We find that nearly all reprogrammed neurons are derived from the neural crest (NC) lineage. Moreover, when rare proliferating NC precursors are selectively ablated, there is a large reduction in the number of reprogrammed neurons. Previous interpretations of this paradigm are that it demonstrates a cell fate conversion across embryonic germ layers (mesoderm to ectoderm). Our interpretation is that this is actually directed differentiation of a neural lineage stem cell in the skin that has intrinsic bias to produce neuronal progeny.

## Highlights

• Neural crest cells are the source of most directly reprogrammed neurons
• The neural crest bias for neuron production is cell autonomous
• Removing neural crest precursors largely reduces reprogramming efficiency
• This challenges the interpretation of directly reprogramming across germ layers

## Teaser

Direct conversion of fibroblast cells into neurons by manipulating gene expression is primarily due to previously unnoticed neural lineage stem cells that are present throughout the skin. Due to the intrinsic bias of this neural cell type to produce neurons, in many cases this may not be the formerly proposed direct conversion of cell types across developmental germ layers.

## Introduction

The field of cellular reprogramming has provided both a challenge to and an update on the process of cellular differentiation and maturation during development. An older view of this process is illustrated by Conrad Waddington’s epigenetic landscape model (Waddington and Conrad, 1957). In this model, a cell early in development has the potential to give rise to progeny that can differentiate into multiple cell types. As development progresses, the differentiation potential of any one precursor becomes more restricted, until finally a fully differentiated and post-mitotic cell is locked into its mature state (Waddington and Conrad, 1957). As demonstrated in the nervous system, subsequent molecular analysis of development has shown that this gradual process of cell lineage restriction and maintenance largely occurs through patterning molecules in the niche, combinatorial expression of transcription factors, and changes to the epigenome (Dessaud et al., 2008; Hobert, 2021; Molyneaux et al., 2007). As a challenge to this, cellular reprogramming has established that ectopic expression of a small number of master regulatory transcription factors in a mature cell can remove it from its locked identity to bring it back to an earlier state in development or directly into another mature cell type. Pluripotent reprogramming has been revealed through overexpression of 4 transcription factors in fibroblast cells, hereby referred to as mouse embryonic fibroblasts (MEFs). A small subset of these MEFs regains pluripotency (termed induced pluripotent stem cells or iPSCs), resembling stem cells of the embryonic inner cell mass (Takahashi and Yamanaka, 2006; Takahashi et al., 2007). In contrast, direct reprogramming involves a direct conversion of one mature cell type into another. This is generally thought to occur without reverting the cell back to an embryonic developmental state and without cell division (Bocchi et al., 2022; Wang et al., 2021). These direct conversions have been shown for a variety of cell types *in vitro* and *in vivo*, including myoblasts, neurons, glia, hepatocytes, cardiomyocytes, and pancreatic exocrine and endocrine cells, and in some cases can occur between cells of different embryonic germ layer lineages (Davis et al., 1987; Guo et al., 2014; Qian et al., 2012; Sekiya and Suzuki, 2011; Tanabe et al., 2018; Vierbuchen et al., 2010; Zhou et al., 2008).

One common feature among various examples of reprogramming is that it is not a fully efficient process, with only a subset of the starting cell population being able to reprogram (Davis et al., 1987; Guo et al., 2014; Qian et al., 2012; Sekiya and Suzuki, 2011; Takahashi and Yamanaka, 2006; Takahashi et al., 2007; Tanabe et al., 2018; Vierbuchen et al., 2010; Zhou et al., 2008). Two broad mechanisms have been proposed to explain this inefficiency in reprogramming: the stochastic and elite (deterministic) models (Hochedlinger and Jaenisch, 2015; Yamanaka, 2009). In the stochastic model, all starting cells have equal potential to reprogram, but only a small subset successfully reprograms due to stochastic or probabilistic mechanisms (Hochedlinger and Jaenisch, 2015; Yamanaka, 2009). In the elite (deterministic) model, a rare subset of the starting cell population has a predetermined intrinsic bias to complete the reprogramming process (Hochedlinger and Jaenisch, 2015; Yamanaka, 2009). Several reports have provided evidence for each of these models, though this has predominantly been done with pluripotent reprogramming and not direct lineage reprogramming (Biddy et al., 2018; Buganim et al., 2012; Hanna et al., 2009; Shakiba et al., 2019; Smith et al., 2010; Yunusova et al., 2017). One other key observation is that often researchers do not investigate the potential heterogeneity in their starting cell population prior to reprogramming. Cells from the embryonic or adult skin are a common substrate for reprogramming due to their ease of collection and culture. This cell culture is regularly described as a homogenous population of dermal MEFs; however, the skin is a highly heterogeneous tissue with multiple stem and progenitor types and developmental lineages (Driskell and Watt, 2015; Rognoni and Watt, 2018; Wong et al., 2006). Using the model of directly reprogramming murine skin cells into neurons (hereby denoted as induced neurons [iNs]), we attempt to answer whether this occurs through a stochastic or elite mechanism and to identify the developmental lineage(s) in the skin that are the substrate(s) for iNs. Through genetic fate mapping and cell-specific ablation experiments, our data indicate that an elite population of skin neural crest (NC) stem and progenitor cells (Seaberg and van der Kooy, 2003), hereby referred to as NC precursors, are almost exclusively the source for iNs. This finding calls into question whether this phenomenon fits the traditional definition of a direct reprogramming, as these skin NC precursors are developmentally derived from the brain, express neural stem cell genes, and have an intrinsic bias to produce neurons under certain culture conditions (Wong et al., 2006).

## Results

### Skin NC cells produce iNs in a cell-autonomous manner

Direct reprogramming experiments were carried out using an established protocol for converting MEFs into iNs (Vierbuchen et al., 2010). To obtain cells for reprogramming, we dissected head and neck skin from embryonic day 14.5 (E14.5) mice and virally delivered and overexpressed the neuron fate-specifying transcription factors BRN2, ASCL1, and MYT1L (BAM factors) (Vierbuchen et al., 2010). After 2 weeks of BAM factor expression, the percentage of iNs in culture is assessed based on the expression of BIII tubulin (TUBB3) and the presence of a process that is at least 3× the length of a small round soma. It is often thought that all the cells in culture are mesodermal-derived MEFs and that the direct conversion of these cells into iNs constitutes a crossing of embryonic lineages from mesoderm to ectoderm (Pang et al., 2011; Vierbuchen et al., 2010). However, as the skin is a heterogeneous tissue, we questioned if there are other types of skin cells in the dish and to what extent they may be substrates for iNs.

**Figure 1:**
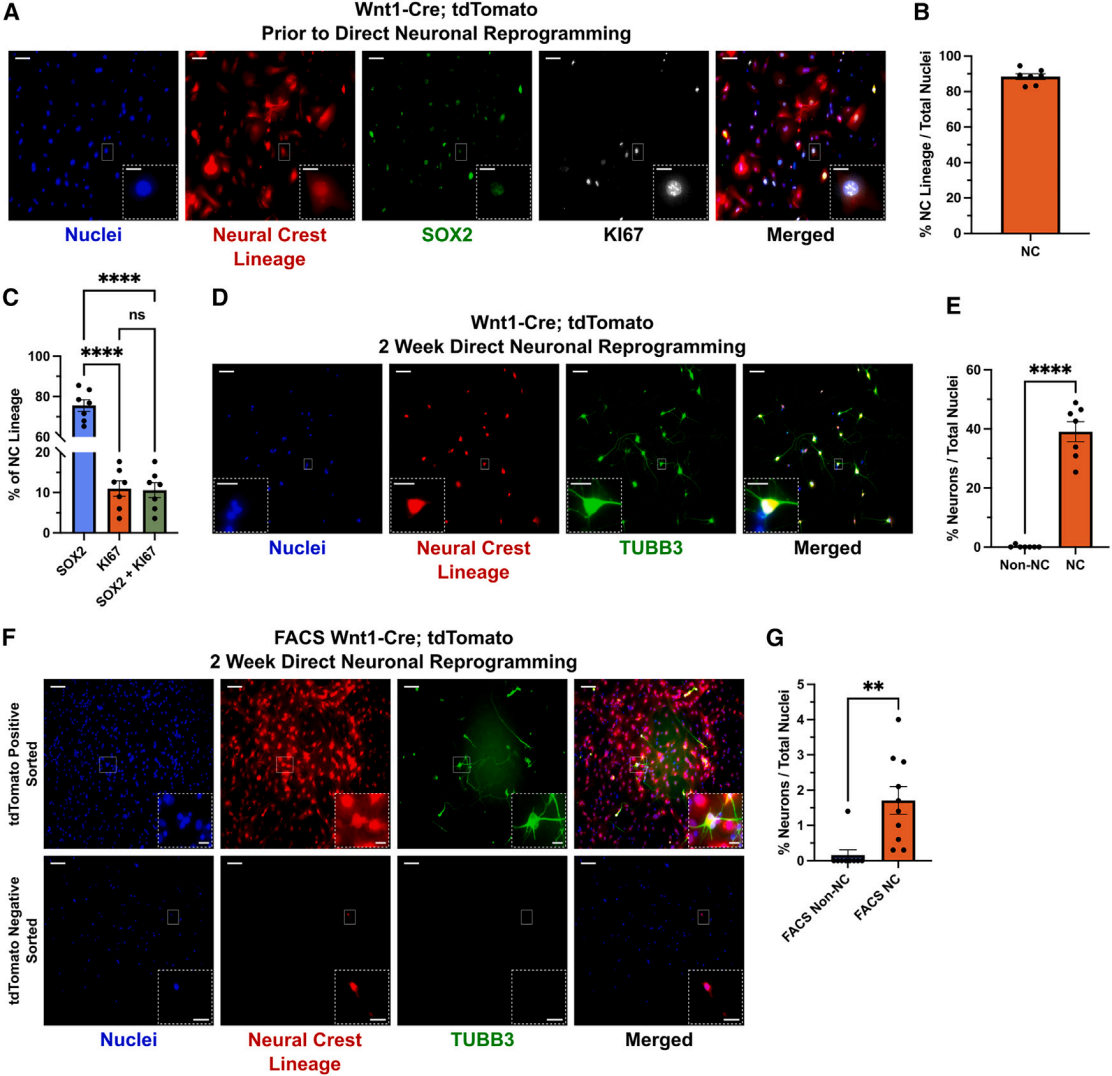
iNs are derived from skin NC cells (A) Representative immunofluorescent micrographs of p3 skin cells derived from e14.5 Wnt1-Cre; tdTomato mice prior to direct iN reprogramming. (B) Quantification of the percentage of total cells that are derived from the NC prior to direct reprogramming (shown in A). *N* = 7 wells from 3 embryos. (C) Quantification of the percentage of total tdTomato-positive cells that are SOX2, KI67, or double-positive prior to direct reprogramming (shown in A). *N* = 7 wells from 3 embryos; one-way ANOVA with Tukey’s multiple comparisons test, ^∗∗∗∗^*p* < 0.0001. (D) Representative immunofluorescent micrographs after 2 weeks of direct iN reprogramming. (E) Quantification of the percentage of total cells that are iNs in the NC and non-NC lineage after 2 weeks of direct iN reprogramming (shown in D). *N* = 7 wells from 3 embryos; two-tailed unpaired Student’s t test, ^∗∗∗∗^*p* < 0.0001. (F) Representative immunofluorescent micrographs of p3 skin cells that were first FACS purified for the presence or absence of tdTomato and then reprogrammed for 2 weeks. (G) Quantification of the percentage of total cells that are iNs in the NC and non-NC sorted fractions (shown in F). Non-NC, *n* = 9 wells from 3 embryos; NC, *n* = 10 wells from 3 embryos; two-tailed unpaired Student’s t test, ^∗∗^*p* = 0.0025. For all micrographs, main scale bar is 100 μm and any insert is 25 μm. Error bars represent mean ± SEM.

We first investigated the NC lineage, which produces multiple cell types in the skin, including melanocytes, Schwann cells, dermal fibroblasts, and adipocytes (Fernandes et al., 2004; Wong et al., 2006). To follow NC-derived cells, we directly reprogrammed dissected skin from Wnt1-Cre; tdTomato mice (Danielian et al., 1998), which indelibly label NC cells early in development prior to their delamination and migration from the neural tube (Danielian et al., 1998; Debbache et al., 2018). Prior to direct reprogramming initiation, skin cells in culture were predominantly derived from the NC and displayed fibroblastic (MEF) morphology (88.43% ± 1.61%; Figures 1A and 1B). This is similar to what our lab has shown previously where the NC makes up ∼60% of cells after three passages and contributes exclusively to cells that reprogram to pluripotency (Shakiba et al., 2019). Many NC MEFs expressed SOX2 (75.56% ± 2.90%; Figures 1A and 1C), confirming the fidelity of our lineage marker and indicating their likely origin from the dermal papilla in the skin (Biernaskie et al., 2009; Driskell et al., 2009; Fernandes et al., 2004; Johnston et al., 2013), while a much smaller NC proportion were proliferating and SOX2-positive (10.61% ± 1.847%; Figures 1A and 1C). As expected from a previous study (Fernandes et al., 2004), these cells being putatively from the derma papilla rarely expressed the NC marker P75, and none of the P75-positive cells were proliferating (Figures S1A and S1B). After direct reprogramming, the percentage of total cells that are iNs are almost entirely tdTomato-positive NC lineage (NC: 39.04% ± 3.38% vs. non-NC: 0.16% ± 0.16%; Figures 1D and 1E). Differences in infectivity of NC and non-NC cells cannot explain the observed iN bias (Figures S1C and S1D), and direct reprogramming does not cause selective survival of NC cells, as there is a slight reduction in NC cells with reprogramming induction when comparted to 2 weeks in culture in the absence of reprogramming induction (Figures S1C and S1E). Under baseline culture conditions, without the induction of BAM factors, NC cells very rarely differentiate into iNs (0.75% ± 0.18%; Figures S1C and S1F). Additionally, after 3 weeks of direct reprogramming, the iNs begin to express more mature markers of excitatory and inhibitory neurons, such as VGLUT1, TBR1, and GABA (Figure S1G). This has previously been observed in the iN reprogramming protocol we are following (Vierbuchen et al., 2010) and confirms the fidelity of our protocol.

There are two potential interpretations as to why NC cells have a bias to produce iNs: either NC cells inhibit non-NC cells from becoming iNs through a cell non-autonomous mechanism or they have a cell-autonomous enhanced ability to directly reprogram to iNs. To test these two models, we sorted cells from Wnt1-Cre; tdTomato mice into pure populations of tdTomato-positive (NC lineage) or negative cells prior to direct reprogramming. After direct reprogramming each of these pure populations, we found that virtually all iNs were present in the tdTomato-positive sorted groups (fluorescence-activated cell sorting [FACS] non-NC: 0.16% ± 0.16% vs. FACS NC: 1.71% ± 0.39%). There were some rare tdTomato-positive cells that escaped into the negative sorted group; however, these were never iNs (Figures 1F and 1G). We would predict many more iNs to be present in the negative sorted groups if the NC was inhibiting these cells from directly reprogramming in mixed (unsorted) experiments. This sorting experiment also argues against the interpretation that the cell culture conditions or expression of BAM factors is causing non-NC skin cells to spuriously turn on the expression of the Wnt1-Cre transgene. In summary, these data strongly suggest a cell-autonomous bias for NC cells in the skin to directly reprogram to iNs.

**Figure 2:**
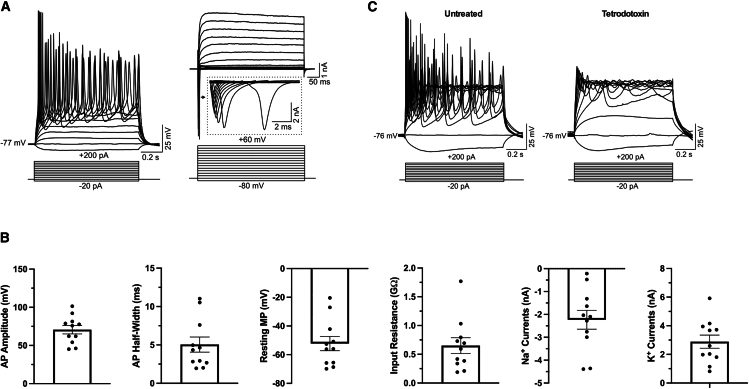
NC lineage iNs display typical electrophysiological properties (A) Representative traces showing the action potentials triggered by injecting a series of current steps from −20 to +200 pA in an NC lineage iN (left). Typical traces displaying the voltage-gated ion currents evoked by stepping the membrane potentials to a series of potentials from −80 to +60 mV in an NC lineage iN (right). Inset displays voltage-gated Na^+^ currents. (B) Histogram showing the amplitude and half-width of action potentials, the resting membrane potentials, input resistance, and voltage-gated Na^+^ (measured at −10 mV) and K^+^ currents (measured at +60 mV) in 11 recorded cells. Error bars represent mean ± SEM. (C) Representative traces showing the evoked action potentials, triggered by injecting a series of current steps from −20 to +200 pA, were blocked by tetrodotoxin at 0.5 μM in an NC lineage iN.

In addition to morphological and molecular characterization, we wanted to ensure that our NC-derived directly reprogrammed iNs display typical functional electrophysiological properties. We performed whole-cell patch-clamp recordings on tdTomato-positive cells (from Wnt1-Cre; tdTomato mice) with the same neuron morphology criteria used for iN efficiency quantification. All cells recorded exhibited characteristic action potentials in response to current injection and voltage-gated sodium and potassium currents (Figures 2A and 2B). Furthermore, action potentials were eliminated by the selective sodium channel blocker tetrodotoxin (Figure 2C). Overall, these data show that iNs derived from NC lineage cells in the skin display the fundamental properties of bona fide neurons.

### MEFs from other regions of the body also display an NC bias in direct iN reprogramming

**Figure 3:**
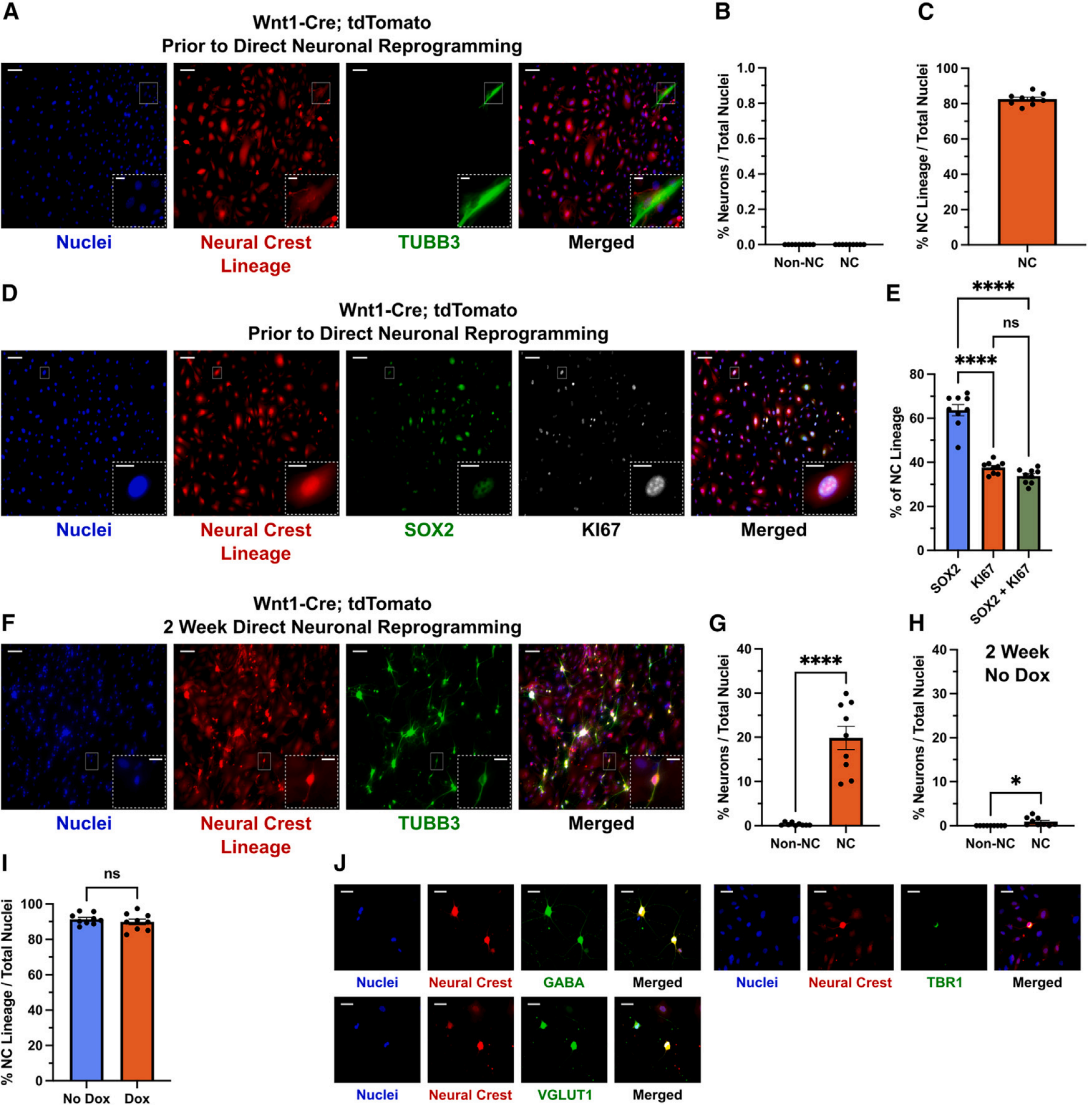
Limb-derived MEFs are predominantly NC lineage and display an NC bias in direct iN reprogramming (A) Representative immunofluorescent micrographs of p3 limb MEFs derived from e14.5 Wnt1-Cre; tdTomato mice prior to direct iN reprogramming. (B) Quantification of the percentage of total cells that are iNs in the NC and non-NC lineage prior to direct reprogramming (shown in A). (C) Quantification of the percentage of total cells that are derived from the NC prior to direct reprogramming (shown in A). (D) Representative immunofluorescent micrographs of proliferating NC lineage MEFs prior to direct reprogramming. (E) Quantification of the percentage of total tdTomato-positive cells that are SOX2, KI67, or double-positive prior to direct reprogramming (shown in D). One-way ANOVA with Tukey’s multiple comparisons test, ^∗∗∗∗^*p* < 0.0001. (F) Representative immunofluorescent micrographs of limb MEFs after 2 weeks of direct iN reprogramming. (G) Quantification of the percentage of total cells that are iNs in the NC and non-NC lineage after 2 weeks of direct reprogramming (shown in F). Two-tailed unpaired Student’s t test, ^∗∗∗∗^*p* < 0.0001. (H) Quantification of the percentage of total cells that are iNs in the NC and non-NC lineage after 2 weeks in culture without the addition of DOX to induce direct reprogramming factor (BAM) expression. Two-tailed unpaired Student’s t test, ^∗^*p* = 0.0109. (I) Quantification of the percentage of total cells that are NC lineage after 2 weeks with or without DOX reprogramming induction. Two-tailed unpaired Student’s t test, *p* = 0.4332. (J) Representative immunofluorescent micrographs of NC lineage limb MEFs after 2 weeks of direct reprogramming expressing excitatory (VGLUT1 and TBR1) and inhibitory (GABA) neuron markers. Scale bar is 50 μm. For all graphs, *n* = 9 wells from 3 embryos. Unless stated, micrograph main scale bar is 100 μm and any insert is 25 μm. Error bars represent mean ± SEM.

We next questioned whether the observed direct iN reprogramming bias in NC dermal MEFs was specific to the region of head and neck skin we were dissecting from, or a more general feature of MEFs derived from other areas of the body. Previous direct iN reprogramming studies have used MEFs dissected from the embryonic arms and legs (Treutlein et al., 2016; Vierbuchen et al., 2010), and so we used this protocol with Wnt1-Cre; tdTomato mice. We observed very similar results to those seen in Figure 1. Prior to direct reprogramming, there were no iNs in culture (Figures 3A and 3B), and most cells were derived from the NC with fibroblastic morphology (82.53% ± 1.090%, Figures 3A and 3C). Within the NC lineage, ∼34% of cells were proliferating SOX2-positive precursors and this comprised nearly all proliferating cells in culture (Figures 3D and 3E). After 2 weeks of direct reprogramming, nearly all iNs were of NC lineage (19.86% ± 2.632% vs. 0.3% ± 0.12%; Figures 3F and 3G). Without direct reprogramming induction (doxycycline), there were very few iNs after 2 weeks (Figure 3H) and no observed bias in the survival of NC cells with reprogramming induction (Figure 3I). Additionally, NC-derived iNs expressed mature inhibitory and excitatory markers (Figure 3J). Therefore, the NC bias in direct iN reprogramming seems to be a general feature of MEFs dissected from multiple regions in the body.

### Epidermal cells rarely directly reprogram to iNs

**Figure 4:**
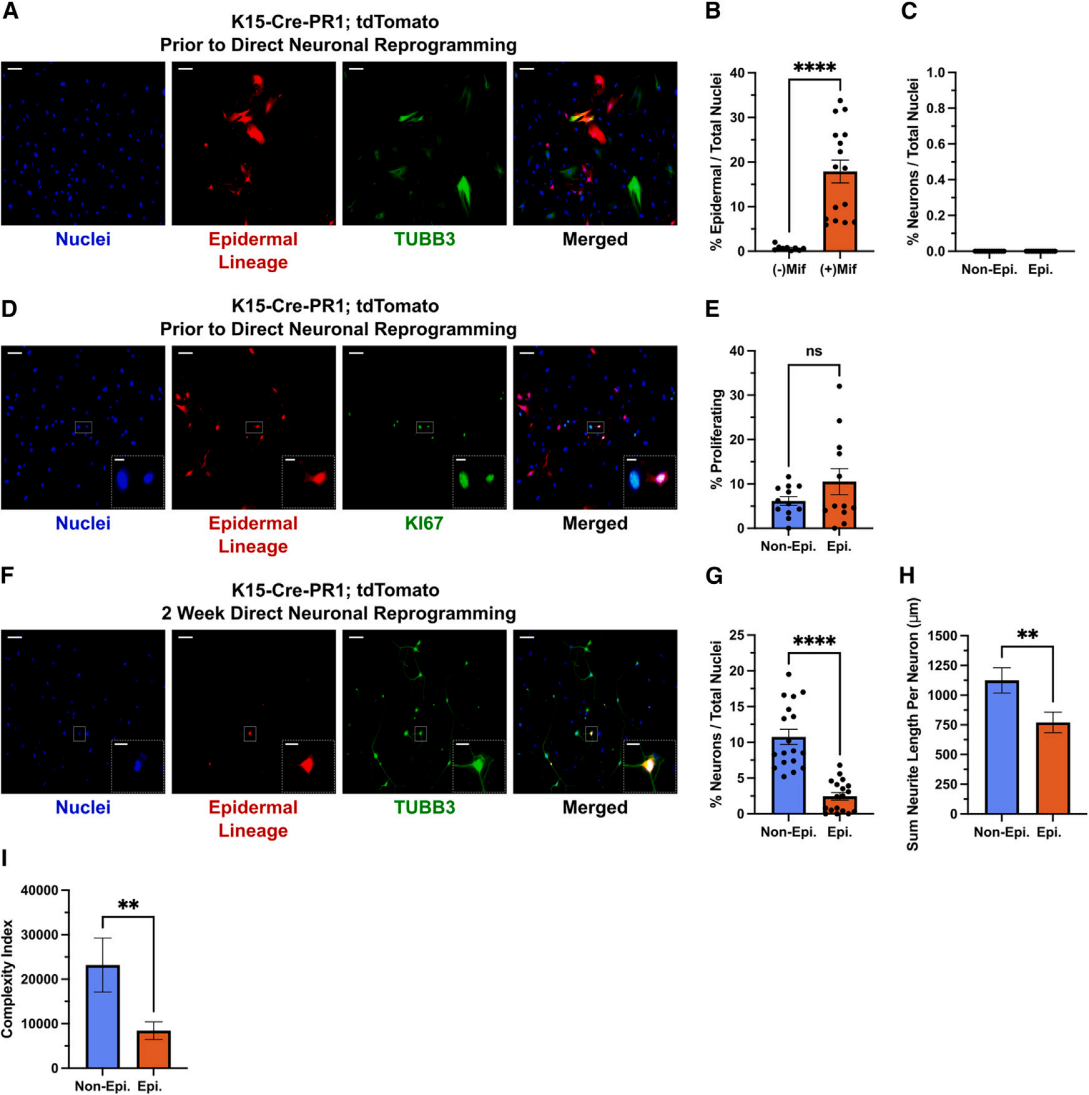
Epidermal lineage cells rarely directly reprogram to iNs (A) Representative immunofluorescent micrographs of p3 skin cells derived from e14.5 K15-Cre^PR1^; tdTomato mice prior to direct reprogramming. Cells shown were treated with mifepristone (Cre inducer) for three passages prior to staining and imaging. (B) Quantification of the percentage of total cells that are epidermal lineage prior to direct reprogramming (shown in A). (−)Mif *n* = 9 wells from 3 embryos, (+)Mif *n* = 16 wells from 5 embryos; two-tailed unpaired Student’s t test; ^∗∗∗∗^*p* < 0.0001. (C) Quantification of percentage of total cells that are iNs prior to direct reprogramming (shown in A). All cells here are treated with mifepristone. *N* = 13 wells from 5 embryos. (D) Representative immunofluorescent micrographs of proliferating skin cells prior to direct reprogramming as described in (A). (E) Quantification of the percentage of non-epi. and epi. cells that are proliferating (shown in D). *N* = 12 wells from 5 embryos; two-tailed unpaired Student’s t test; *p* = 0.1737. (F) Representative immunofluorescent micrographs of mifepristone-treated skin cells after 2 weeks of direct reprogramming as described in (A). (G) Quantification of the percentage of total cells that are iNs in the epidermal and non-epidermal lineages (shown in F). *N* = 18 wells from 5 embryos; two-tailed unpaired Student’s t test; ^∗∗∗∗^*p* < 0.0001. (H) Quantification of the total length of all neurites per iN (shown in F). Mann-Whitney test; ^∗∗^*p* = 0.0074. (I) Quantification of complexity index per iN (shown in F). Mann-Whitney test; ^∗∗^*p* = 0.0012. (G and H) Non-epi. *n* = 95 neurons; epi. *n* = 65 neurons. For all micrographs, main scale bar is 100 μm and any insert is 25 μm. Error bars represent mean ± SEM.

Given that NC cells do not make up the entirety of the skin culture, we investigated what other types of cells may be present and to what extent are they able to directly reprogram to iNs. Skin was dissected from a drug-inducible K15-Cre^PR1^; tdTomato mouse line at E14.5 (Morris et al., 2004). *K15* is a marker of epidermal precursors that give rise to all cell types of the cutaneous epithelium (epidermis, hair follicles, and sebaceous glands) (Morris et al., 2004; Troy et al., 2011); therefore, cells comprising the epidermal layer will be marked with tdTomato in culture. Prior to initiating direct iN reprogramming, epidermal lineage cells made up a minor percentage of total cells in culture (17.89% ± 2.571%; Figures 4A and 4B), and there was low background expression without mifepristone Cre induction (0.69% ± 0.19%; Figure 4B). While some cells in culture expressed TUBB3, these cells all displayed fibroblastic morphology and did not meet the criteria for iN classification (Vierbuchen et al., 2010) (Figures 4A and 4C). Of the epidermal cells, some were putative proliferating precursors prior to direct reprogramming, and these were not significantly different in relative amount compared to the proliferating cells in the non-epidermal (presumptive NC) lineage (non-epi. 6.15% ± 1.00% vs. epi. 10.51% ± 2.93%; Figures 4D and 4E). Infected cells in culture for 2 weeks in neuron-permissive media without doxycycline induction of BAM factors also displayed almost no iNs (Figures S2A and S2B). After 2 weeks of direct reprogramming, the percentage of total iNs was almost all tdTomato-negative non-epidermal cells (non-epi. 10.76% ± 1.07% vs. epi. 2.45% ± 0.52%; Figures 4F and 4G). This difference in reprogramming efficiency could not be explained by a difference in infectivity between epidermal and non-epidermal cells, as both groups showed a >95% transduction efficiency when analyzed after 2 weeks in culture (Figures S2A and S2C). It is possible that forced expression of BAM factors selectively kills off epidermal cells; however, after direct reprogramming, the percentage of epidermal cells was similar to pre-reprogramming and to 2 weeks without BAM factor induction (Figure S2D). In observing the morphology of iNs, there was a reduction in both the length and complexity index (Pillai et al., 2012) of neurites from epidermal cells compared to non-epidermal (NC) lineage cells (Figures 4H and 4I). These data suggest that in the rare occasions where direct iN reprogramming occurs from an epidermal cell, the resulting iNs cannot mature as fully as from presumptive NC lineage cells. It is notable that there are more epidermal lineage reprogrammed iNs that would be expected from the NC lineage experiments in Figure 1. Given that most tdTomato-positive cells (∼90%) are not proliferating (Figures 4D and 4E) and only ∼40% of tdTomato-positive cells stain for the post-mitotic epidermal marker K10 (Kartasova et al., 1993) (Figures S2E and S2F), it is possible that our K15-Cre^PR1^; tdTomato reporter mouse is non-specifically marking some cells in culture and we are overestimating the comparatively infrequent epidermal iN events (Figures S2E and S2F). Despite this, the fact that there are morphological differences between epidermal and non-epidermal lineage iNs does suggest that, in rare instances, an epidermal cell can be directly reprogrammed into an iN. In summary, these data show that cells from the epidermis persist in standard iN reprogramming culture conditions but very rarely can directly reprogram to an iN.

### Selectively ablating NC precursors substantially reduces iN reprogramming efficiency

**Figure 5:**
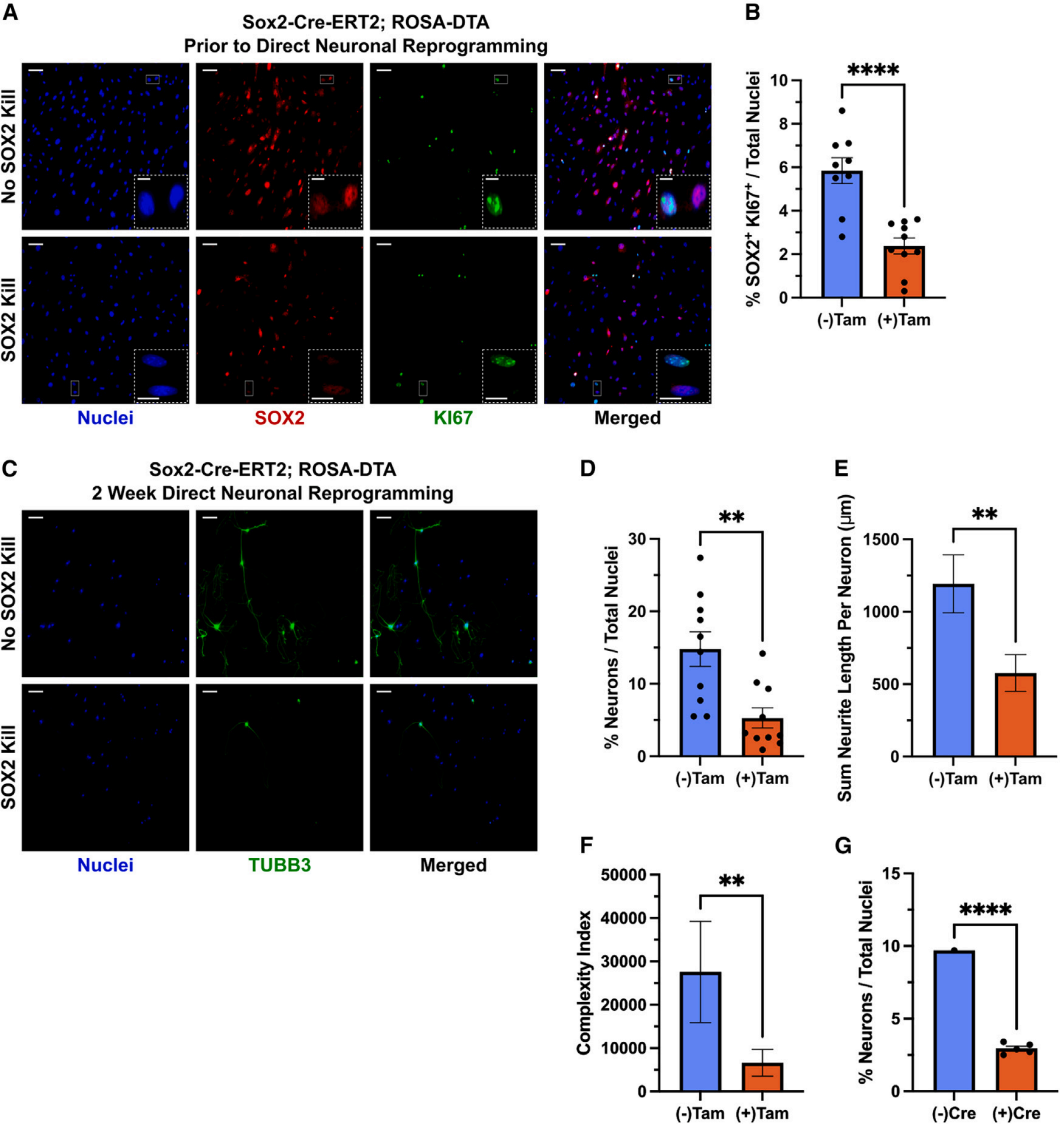
NC precursor cells are the source of most iNs (A) Representative immunofluorescent micrographs of p3 skin cells derived from e14.5 Sox2-Cre^ERT2^; ROSA-DTA mice prior to direct reprogramming. (B) Quantification of the percentage of total cells that are NC precursors (SOX2 and KI67 positive) as shown in (A). (−)Tam cells are cultured without tamoxifen (no SOX2 kill) and (+)Tam cells are treated 3 days with tamoxifen (SOX2 kill). (−)Tam, *n* = 9 wells; (+)Tam, *n* = 10 wells; from 3 embryos; two-tailed unpaired Student’s t test, ^∗∗∗∗^*p* < 0.0001. (C) Representative immunofluorescent micrographs of Sox2-Cre^ERT2^; ROSA-DTA skin cells after 2 weeks of direct reprogramming. (D) Quantification of the percentage of total cells that are iNs after 2 weeks of direct reprogramming with or without prior tamoxifen treatment (shown in C). (−)Tam, *n* = 10 wells; (+)Tam, *n* = 10 wells; from 4 embryos; two-tailed unpaired Student’s t test, ^∗∗^*p* = 0.0030. (E) Quantification of the total length of all neurites per iN (shown in C). Mann-Whitney test; ^∗∗^*p* = 0.0061. (F) Quantification of complexity index per iN (shown in C). Mann-Whitney test; ^∗∗^*p* = 0.0049. (E and F) (−)Tam, *n* = 45 neurons; (+)Tam, *n* = 34 neurons. (G) Quantification of the percentage of total cells that are iNs after 2 weeks of direct reprogramming with or without prior tamoxifen treatment *in vivo*. Two-tailed unpaired Student’s t test, ^∗∗∗∗^*p* < 0.0001. (−)Cre, *n* = 1 well from 4 pups; (+)Cre, *n* = 5 wells from 3 pups. For all micrographs, main scale bar is 100 μm and any insert is 25 μm. Error bars represent mean ± SEM.

In the skin, there are NC-derived precursors that, when removed from their *in vivo* niche, can differentiate into multiple ectodermal (including neurons) and mesodermal cell types and extensively self-renew (Biernaskie et al., 2009; Fernandes et al., 2004; Johnston et al., 2013; Wong et al., 2006). These NC precursors express the neural stem cell marker *Sox2*, can be found below the hair follicle in the dermal papilla or associated with hair follicle nerve terminals, and have *in vivo* roles in regulating hair follicle formation and dermal wound repair (Biernaskie et al., 2009; Fernandes et al., 2004; Johnston et al., 2013; Wong et al., 2006). Therefore, we examined to what extent NC precursors might be the specific substrate cell for directly reprogrammed iNs. To do this, we directly reprogrammed skin cells from Sox2-Cre^ERT2^; ROSA-DTA mice (Wu et al., 2006), where, upon induction of Cre with tamoxifen, *Sox2*-expressing NC precursor cells will be ablated with expression of the diphtheria toxin A chain. Prior to direct reprogramming, and after three consecutive days of tamoxifen administration, there was a large (∼60%) reduction in the number of proliferating (KI67-positive) NC precursors ((−)Tam: 5.94% ± 0.59% vs. (+)Tam: 2.38% ± 0.37%; Figures 5A and 5B). Some NC cells also maintained SOX2 protein presence (at least temporarily) after exiting the cell cycle (Figure 5A). As with the NC and epidermal lineage experiments, prior to direct reprogramming, there were cells that expressed TUBB3 with fibroblastic (MEF) morphology, but no bona fide iNs (Figures S3A and S3B). Using ROSA-DTA mice without Cre, tamoxifen alone did not have any effect on proliferating NC precursors (Figures S3C and S3D). When cells were directly reprogrammed after 3 days of tamoxifen pretreatment, there was a ∼64% decrease in the percentage of iNs ((−)Tam: 14.80% ± 2.39% vs. (+)Tam: 5.30% ± 1.40%; Figures 5C and 5D). While there was not a complete absence of iNs with tamoxifen pre-treatment, the remaining iNs showed a reduction in neurite length and complexity index (Figures 5E and 5F). Some iNs may be present due to incomplete tamoxifen-induced Cre recombination efficiency, but the neuronal morphology also indicates that either more mature (non-precursor) NC cells or epidermal cells cannot fully complete iN reprogramming and mature as effectively. Tamoxifen exposure did not affect the cell’s ability to be infected or the baseline numbers of iNs without doxycycline BAM factor induction over the 2-week protocol (Figures S4A–S4C). In addition, tamoxifen in no-Cre ROSA-DTA controls did not affect the ability of cells to directly reprogram to iNs (Figures S4D and S4E). Previous direct iN reprogramming work also has shown that there is not an initial proliferative period in the cells that successfully reprogram, and that these cells must go post-mitotic almost immediately upon BAM factor expression (Vierbuchen et al., 2010). We reproduced this result through exposing cells regularly to EdU over the 2-week direct reprogramming period. Without tamoxifen pretreatment, there was a low percentage (7.30% ± 4.09%) of iNs that were co-labeled with EdU and with tamoxifen treatment there were no iNs co-labeled (Figures S4F). Further, we wondered whether killing off postnatal SOX2-positive skin NC precursors *in vivo* (using a tamoxifen cream applied to the skin) prior to dissection and culture would have similar effects to our *in vitro* SOX2 kill experiments (Vasioukhin et al., 1999). Indeed, we see a massive reduction (∼80%) in the efficiency of iN reprogramming when NC precursors are ablated from early postnatal skin *in vivo* (Figure 5G). These data suggest that our results in Figures 1, 2, 3, and 4 are not purely a phenomenon of embryonic MEFs and that cell culture conditions are not creating an artificial NC precursor state only gained *in vitro*. Lastly, it is significant that the percent reduction in NC precursors (Figure 5B) very closely resembles the percent reduction in iNs (Figure 5D). This observation promotes confidence in the idea that the source of most iNs is a proliferating NC precursor cell.

## Discussion

**Figure 6:**
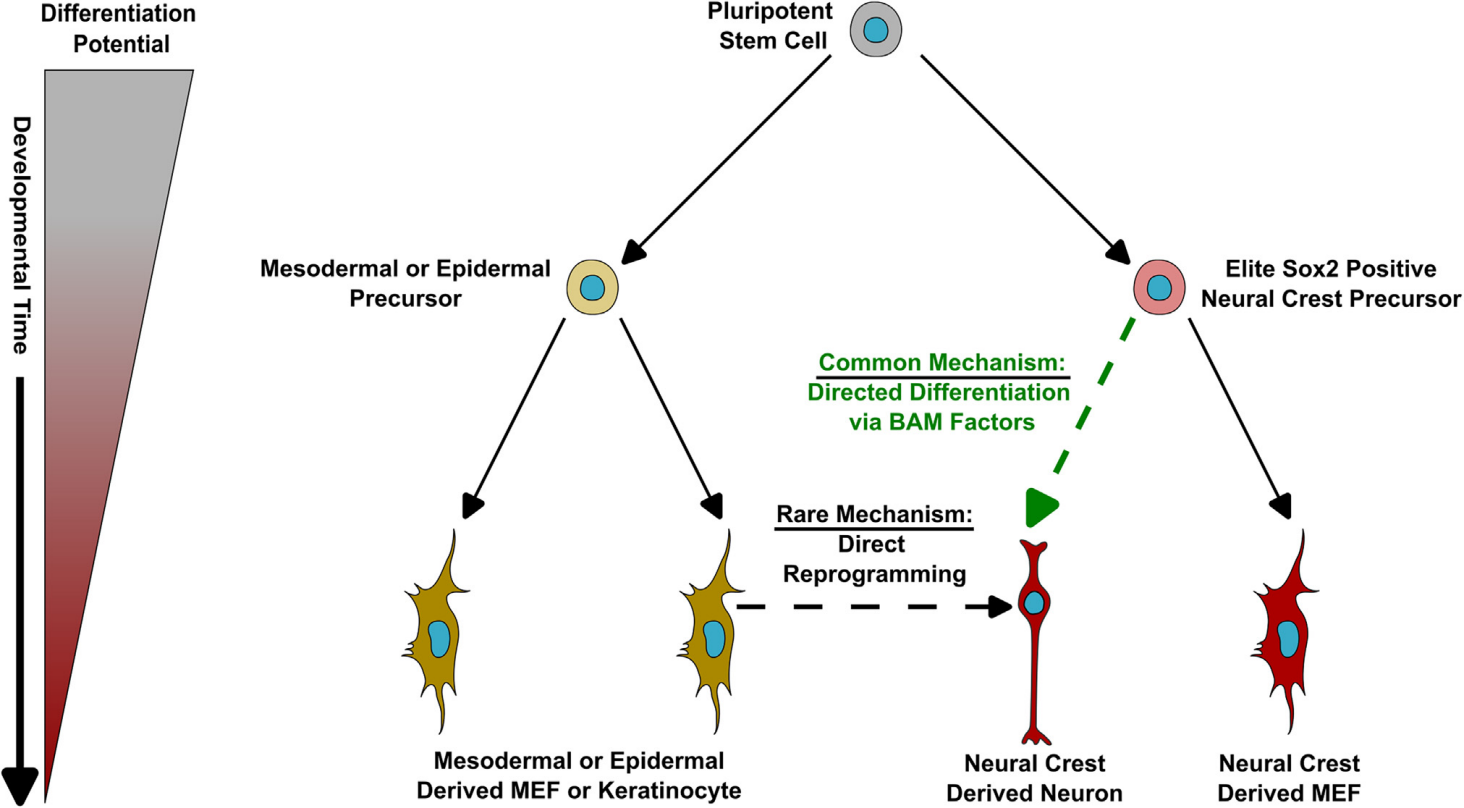
Updated model of how iNs arise in direct reprogramming experiments

The experiments described here present an alternative explanation for the findings of direct reprogramming of functional iNs from MEFs (Figure 6). This phenomenon was originally thought to be an early example of a direct conversion between cells from different embryonic germ layers (mesodermal MEFs to neuroectodermal neurons) and to not require the starting cells to go through a stem or progenitor state (Bocchi et al., 2022; Treutlein et al., 2016; Vierbuchen et al., 2010; Wang et al., 2021). Our data challenge both assumptions in that nearly all iNs are derived from the NC, which shares a developmental germ layer origin with neurons, and that NC precursor cells are the likely source of most iNs from multiple MEF-containing regions of the body. We employed a transgenic fate mapping strategy to determine the extent of cell type heterogeneity that is present in cell cultures typically used in direct or pluripotent reprogramming experiments. As our lab has previously reported in relation to pluripotent reprogramming (Shakiba et al., 2019), we find that NC cells dominate after 3 passages prior to the direct reprogramming period. We show as well that there are some epidermal cells prior to direct reprogramming that persist throughout the reprogramming period. We are likely overestimating the number of epidermal cells in culture and their iN reprogramming efficiency based on staining with additional epidermal markers, but even with this discrepancy, NC and epidermal cells can account for all lineages in culture. The observed bias for NC origin of iNs cannot be explained alone by their high numbers prior to reprogramming. This was confirmed through FACS experiments purifying non-NC skin cells, where only exceedingly rare iNs were observed. The FACS experiments also indicate that the NC bias in direct reprogramming is cell autonomous and not due to NC inhibiting other cell types from reprogramming. There was considerably lower iN efficiency in the FACS experiments compared to unsorted and mixed experiments; however, there are previous reports that neural lineage stem cells undergo increased cell death and cellular stress response after being run through an FACS machine (Bowles et al., 2019; Coles et al., 2021). We predict that either increased death or stress of NC precursors is resulting in lower iN efficiency in these experiments. Despite this lower efficiency, the overall trend of a bias in iN reprogramming is consistent among sorted and unsorted experiments. A small proportion of epidermal lineage cells were able to successfully convert to iNs; however, these iNs were less morphologically mature, presenting not only quantitative differences in iN reprogramming efficiency but also qualitative differences in rare iNs that do arise from epidermal cells. As epidermal cells share an embryonic ectodermal origin with neurons (Muñoz-Sanjuán and Brivanlou, 2002), these epidermal conversions still do not represent direct reprogramming across germ layers as previously proposed (Vierbuchen et al., 2010). There were proliferating precursors in the epidermal and NC lineages prior to direct reprogramming, disputing proliferating precursor presence in a certain lineage alone as a factor in reprogramming bias. Our results also argue against a survival bias for NC cells in direct reprogramming conditions, as there were equivalent numbers of epidermal lineage cells prior to and after reprogramming. Thus, forced expression of BAM factors does not seem to be selectively killing off non-NC cells. These data all point to the existence of an intrinsic barrier to direct cell type conversion in non-NC cells and that the ease of crossing this barrier previously was overestimated (Bocchi et al., 2022; Wang et al., 2021).

NC precursor cells exist in many tissues throughout the body and have a multipotential differentiation profile that is wider ranging that any other adult stem cell type (Achilleos and Trainor, 2012). In the skin, there are NC precursors that express the canonical neural stem cell marker *Sox2*. When taken out of their inhibitory *in vivo* niche and placed under specific culture conditions, they can extensively self-renew and give rise to neural cell types including neurons, glia, and myelinating and non-myelinating Schwann cells (Biernaskie et al., 2009; Fernandes et al., 2004; Johnston et al., 2013; Wong et al., 2006). We show that a small percentage of cells in culture are proliferating SOX2-positive NC precursors. As we are dissecting skin from E14.5, it is likely that the *in vivo* origin of these NC precursors is the dermal condensates underneath the developing hair follicle (Driskell et al., 2009). There are both NC-derived Schwann cell precursors and melanocyte precursors (melanoblasts) present in the skin that possess stem cell properties, including self-renewal and a broad multipotential differentiation profile (Motohashi et al., 2009; Solovieva and Bronner, 2021; Wong et al., 2006). Despite this, it is unlikely that these NC cell types are the source of iNs in our culture. As described, our proliferating NC precursors do not express P75, which is a marker for both Schwann cell precursors and melanoblasts (Motohashi et al., 2009; Solovieva and Bronner, 2021; Wong et al., 2006). Furthermore, Schwann cell precursors require axon-derived growth factors (not present in our growth medium) to survive in culture (Dong et al., 1995). In the future, it will be useful to purify these other NC precursor cell types in permissive culture conditions to determine if elite reprogramming ability is a general feature of all NC precursor subtypes in the skin, or specific to those of the dermal papilla. When NC precursors are ablated *in vitro* prior to direct reprogramming, we see a substantial reduction in the efficiency of iNs. It is compelling that the percentage of proliferating NC precursors we see prior to direct reprogramming is similar to the percentage of iNs, and the corresponding reduction in iN efficiency matches the reduction in proliferating NC precursors. The fact that there is not a complete reduction in iNs may be due to inefficient Cre induction and that some post-mitotic NC cells may also be able to complete iN reprogramming. Like the epidermal lineage experiments, iNs still present from potential downstream NC cells had reduced morphological maturity. When SOX2-positive NC cells are depleted from the early postnatal skin *in vivo*, we also see similar reductions in iNs. Direct iN reprogramming across germ layers also has been proposed from human dermal skin fibroblasts (Pang et al., 2011). Given that NC precursors with self-renewal capability and broad multipotential differentiation profiles exist up to old age in human skin (Moghadasi Boroujeni et al., 2019), it seems likely that NC precursors are also the source of human iNs. Altogether, the NC precursor ablation experiments support an elite model over a stochastic model of direct reprogramming (Yamanaka, 2009). We can identify prospectively a rare population of cells with an intrinsic bias for direct reprogramming, which, when removed, eliminate most supposedly reprogrammed iNs.

In contrast to previous interpretations of direct iN reprogramming from MEFs (Vierbuchen et al., 2010), our explanation for the emergence of most iNs in this paradigm is that intrinsically biased NC precursors (when expressing BAM transcription factors) undergo directed neuronal differentiation, rather than direct lineage conversion of a non-neural MEF (Figure 6). Going forward, it will be important to understand the underlying mechanisms by which NC precursors can differentiate into neurons when exposed to iN reprogramming factors. There is a recent collection of studies that show NC precursors either maintain or reactivate a pluripotent gene expression signature previously thought to only exist in pre-gastrulation pluripotent epiblast cells. These studies also suggest that this NC pluripotent signature is necessary for this lineage’s broad differentiation potential (Buitrago-Delgado et al., 2015; Pajanoja et al., 2023; Scerbo and Monsoro-Burq, 2020; Schock et al., 2023; Zalc et al., 2021). Some NC precursors in mature tissues (including the skin), when removed from their *in vivo* niche, retain the broad differentiation profile (*in vitro* and upon transplantation *in vivo*) that is seen in embryonic development prior to migration (Achilleos and Trainor, 2012). Therefore, it is possible that NC precursors in our culture conditions reactivate some components of this pluripotent signature. We speculate that this creates a permissive chromatin state that allows for BAM factors to initiate and maintain a neuronal differentiation program. Indeed, our lab has previously shown that the NC is the primary substrate for pluripotent reprogramming (Shakiba et al., 2019), and this may also be due to a partial reacquisition of a pluripotent signature prior to delivery of Yamanaka factors. Additionally, a study looking at reprogramming pericytes (which can be NC derived (Yamanishi et al., 2012)) to iNs found a neural stem cell-like gene signature when analyzing both pericytes and MEF starting cell gene expression prior to iN reprogramming (Karow et al., 2018). Our data predict that this neural stem cell-like gene expression signature in MEFs is due to NC precursors. The ability to chemically reprogram fibroblasts to neurons is also in accordance with our hypothesis in that some of the chemical components are involved in the differentiation of neural stem cells during brain development (Li et al., 2015).

There seemingly are incontrovertible examples of direct iN reprogramming across embryonic germ layers from hepatocytes or blood cells to iNs (Marro et al., 2011; Tanabe et al., 2018); however, our study highlights that, in many cases, NC precursors may be the source of the desired neuron cell type. In the case of reprogramming hepatocytes to neurons (Marro et al., 2011), there is some evidence that NC-derived pericytes can differentiate into hepatocyte-like cells (Sierra et al., 2020). It will therefore be important to check this using NC lineage mice in hepatocyte to neuron direct reprogramming and to FACS purify hepatocytes prior to reprogramming. Another intriguing possibility is rare cell fusion with an NC cell, as fusion has been shown to influence pluripotent reprogramming (Silva et al., 2006). In the example of neuronal reprogramming with T cells (Tanabe et al., 2018), it will be essential to use an NC lineage mouse to ensure that cell fusion with the NC is not a contributing factor. As NC cells are widely distributed in many tissue types throughout the body (Erickson et al., 2023), we predict that they may be unnoticed in the starting cell populations and contribute to the desired cell type in numerous reprogramming paradigms. There is even recent evidence in direct lineage conversion of MEFs to endodermal progenitors that the small number of identifiable cells that can complete the process initially expresses some components of the pluripotency gene network (Kamimoto et al., 2023). As aforementioned, we predict that this is due to a pluripotency gene-expressing NC precursor being the substrate for this differentiation. In summary, this work and our previous results with pluripotent reprogramming (Shakiba et al., 2019) raise the possibility that an elite NC reprogramming mechanism in the starting cell population may be a broader feature of direct and developmental reprogramming.

## Experimental procedures

### Mice

All use of animals was approved by the University of Toronto Animal Care Committee in accordance with the Canadian Council on Animal Care. Animals were housed in the Division of Comparative Medicine facility at the University of Toronto and kept on a 12-h light/dark cycle with *ad libitum* feeding. For studies using mouse embryos, mice were bred at 6–12 weeks of age and plugs were checked every morning to determine the approximate time of conception. The following mouse strains were purchased from Jackson Laboratory: Wnt1-Cre (003829), K15-Cre^PR1^ (005249), Sox2-Cre^ERT2^ (017593), ROSA-tdTomato (007914), and ROSA-DTA (010527).

### Primary cell isolation and culture

Cell dissection was performed as previously reported (Shakiba et al., 2019). Briefly, E14.5 embryos were obtained from pregnant dams, and head + neck region was removed from embryos using curved forceps and placed in a Petri dish with cold artificial cerebrospinal fluid (Reynolds and Weiss, 1992). Cells dissected from each embryo were kept separate throughout the entire procedure. Using curved microdissection scissors, a region of skin was cut that included the head continuing to the back of the neck. Special care was taken to cut skin above the eyes and otic placodes to avoid neural tissue contamination. Skin was then peeled off using curved forceps and further cut into smaller pieces in the dish. Pieces of skin were placed in 0.25% trypsin-EDTA (Thermo, 25200056) for 10 min at 37°C. Tissue was spun down at 0.4 rcf for 5 min and resuspended in 1.5 mL of feeder media, which includes DMEM (Thermo, 11995065) + 10% FBS (Gibco, ESC qualified) + 0.5× penicillin-streptomycin (Thermo, 15070063). Mechanical dissociation with a fire-polished glass pipette followed by passage through a 40 μm cell strainer created a single-cell suspension. Before plating, 10 cm dishes (Thermo, 150460) were coated with 0.1% gelatin (STEMCELL Technologies, 07903) for 30 min at room temperature. For experiments in Figure 3, cells were dissected from arms and legs rather than head and neck region, as previously reported (Vierbuchen et al., 2010). Briefly, arms and legs were removed below the shoulder and hip joints and minced thoroughly with curved microdissection scissors. After this, cells were processed as described in the head and neck dissection earlier. The entire contents from one embryo were seeded in each 10 cm dish and cultured in feeder media. Media was replaced every 3 to 4 days and cells were passaged at 1:3 when they reached 70%–90% confluency. Passage was done using 0.25% trypsin-EDTA (37°C, 5 min). Cells were passaged three times before starting direct reprogramming. For experiments using K15-Cre^PR1^; tdTomato, cells were fed with DMEM + 10% FBS + P/S + 100 nM mifepristone (Sigma, M8046) every 3 to 4 days prior to direct reprogramming initiation.

### Direct neuronal reprogramming

Passage three skin cells were plated at either 100,000 cells per well (in 2.5 mL of media) in a 6-well plate (for FACS and *in vivo* experiments) or 20,000 cells per well (in 500 μL of media) in a 24-well plate (for all other experiments) in DMEM + 10% FBS + P/S. Before seeding, wells were coated with ready-to-use hESC-qualified Geltrex (Thermo, A1569601) or a 1:30 dilution of growth factor-reduced Matrigel (Corning, 354230) for 1 h at 37°C. For NC stem and progenitor cells ablation experiments (Sox2-Cre^ERT2^; ROSA-DTA), cells were treated with 1 μM (Z)-4-hydroxytamoxifen (Sigma, 508225) in feeder media every day for 3 consecutive days. 0.1 mL (∼2 MOI per virus) of each virus (Brn2, Ascl1, Myt1l, rtTA, eGFP, totaling 0.5 mL) in feeder media with 8 μg/mL polybrene (Sigma TR-1003) was added to the skin cells. The next day, cells were exchanged with fresh feeder media with 2 μg/mL doxycycline (Sigma, D9891) to induce expression of the transduced reprogramming factors. 48 h later, media was switched to a serum-free neuronal differentiation and maturation media (SFM). This SFM includes a 1:1 mixture of DMEM (Thermo, 12100046) and F12 (Thermo, 21700075) at a final 1× concentration, 0.6% D-glucose (Sigma, G6152), 5 mM HEPES (Thermo, 15630080), 3 mM NaHCO_3_ (Thermo, 25080094), 2 mM L-glutamine (Thermo, 25030081), 25 μg/mL insulin (Sigma, I5500), 100 μg/mL apo-transferrin (Sigma, T2252), 20 nM progesterone (Sigma, P6149), 60 μM putrescine (Sigma, P5780), and 30 nM sodium selenite (Sigma, S9133). 500 μL of SFM was changed every 2 to 3 days and supplemented fresh with 1× *N*-2 (Thermo, 17502048), 1× B-27 (Thermo, 17504044), and 2 μg/mL doxycycline. Note that some conditions contain the supplemented SFM without doxycycline (no direct reprogramming induction). From the addition of doxycycline (day 0), direct neuronal reprogramming was carried out for 2 weeks total. For the EdU experiments, the aforementioned SFM was additionally supplemented with 10 μM EdU (Thermo A10044) throughout the direct reprogramming period.

### Statistical analysis

Graphs were created and statistical tests were performed using Prism 9 software. For all graphs, dots represent individual wells and error bars represent mean ± SEM. Replicate number and statistical tests used, and results of those tests are present in each figure legend.

### Additional procedures

Please see supplemental document for additional experimental procedure information on genotyping, viral production, FACS, *in vivo* NC precursor ablation, immunocytochemistry, microscopy and image quantification, and electrophysiology.

## Resource availability

### Lead contact

Further information and requests for resources and reagents should be directed to and will be fulfilled by the lead contact, Justin Belair-Hickey (justin.belair.hickey@mail.utoronto.ca).

### Materials availability

This study did not generate new unique reagents. However, any questions about reagents or animals used can be directed to the lead contact.

### Data and code availability

This study did not generate any new datasets or code. Any inquiries about image collection and quantification can be made to the lead contact.

## Acknowledgments

We thank all members of the van der Kooy laboratory for their helpful discussion of the concepts, data, and comments on the manuscript and Yongqian Wang for figure design assistance. This work was supported by a 10.13039/100022706CIHR Foundation Grant (FDN: 148407 to D.v.d.K.), Medicine by Design Program (TIPS:CFREF C1TPA-2016-0 to D.v.d.K.), 10.13039/501100000024CIHR (grant FDN: 154336 to M.W.S.), and the 10.13039/501100004089Krembil Foundation (to M.W.S.).

## Author contributions

J.J.B.-H. designed and carried out the experiments, analyzed the data, and wrote the paper. A.F. designed and carried out the experiments and analyzed the data. R.S.S. designed and carried out the experiments and analyzed the data. W.Z. designed and carried out the experiments and analyzed the data. M.W.S. designed the experiments and analyzed the data. B.L.K.C. provided technical assistance with dissections. D.v.d.K. designed the experiments, analyzed the data, and wrote the paper.

## Declaration of interests

The authors declare no competing interests.

## Supplemental information

Document S1. Figures S1–S4, Table S1, and supplemental experimental procedures

Document S2. Article plus supplemental information
