# Supplementary material for: Neural crest precursors from the skin are the primary source of directly reprogrammed neurons
Source: Stem Cell Reports. 2024 Oct 31;19(11):1620–34. doi: 10.1016/j.stemcr.2024.10.003 (PMC11589197; doi:10.1016/j.stemcr.2024.10.003)
Supplement: Document S1. Figures S1–S4, Table S1, and supplemental experimental procedures [file mmc1.pdf]

Stem Cell Reports, Volume *19*

Supplemental Information

Neural crest precursors from the skin are the primary source of directly reprogrammed neurons

Justin J. Belair-Hickey, Ahmed Fahmy, Wenbo Zhang, Rifat S. Sajid, Brenda L.K. Coles, Michael W. Salter, and Derek van der Kooy

Supplemental Figures and Legends

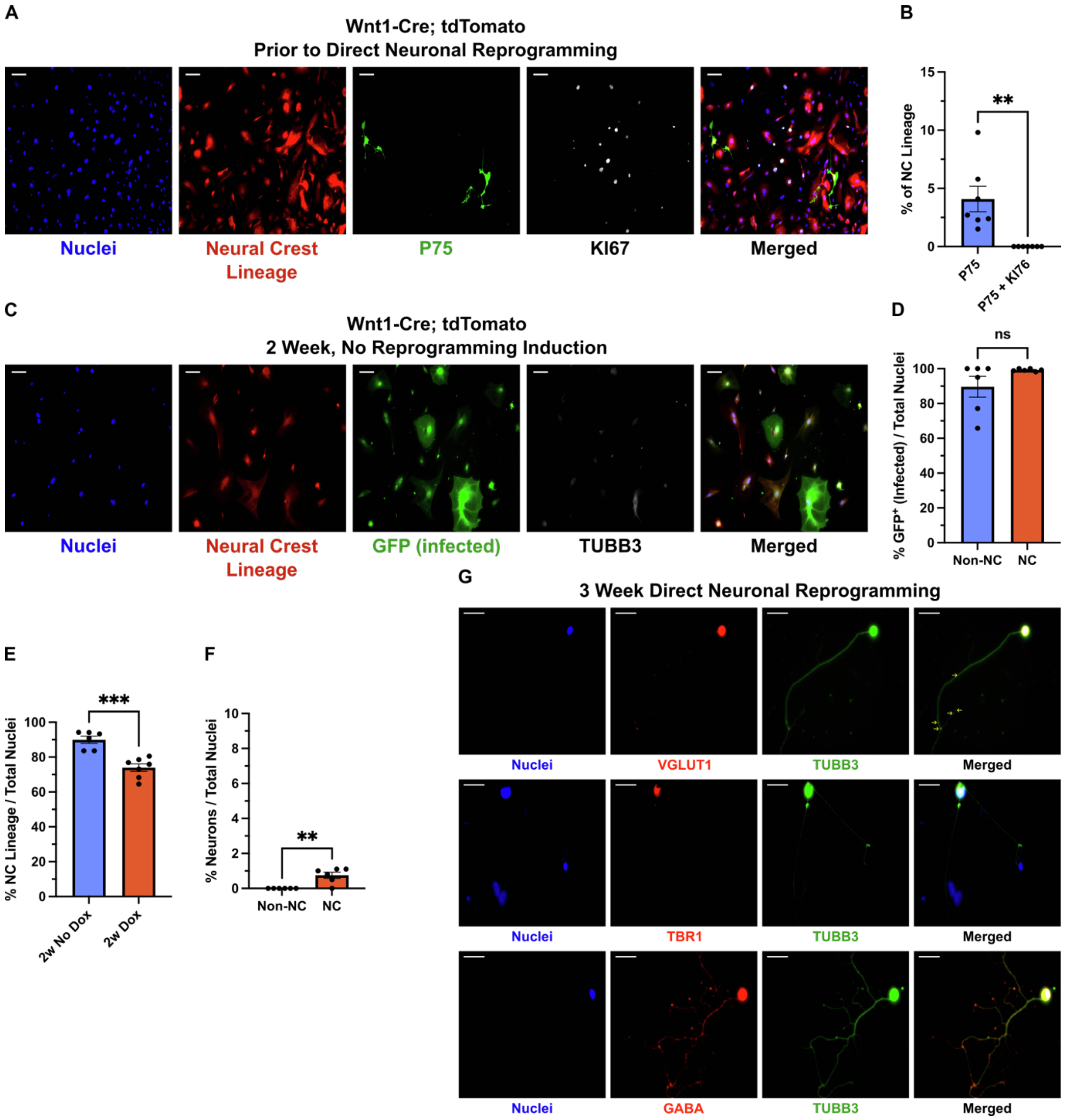

Figure S1 NC transduction efficiency, NC cell number, iNs without reprogramming factor induction, and excitatory and inhibitory neuron markers.
(A) Representative immunofluorescent micrographs of p3 skin cells derived from e14.5 Wnt1-Cre; tdTomato mice prior to direct iN reprogramming. (**B**) Quantification of the percentage of total tdTomato cells that are P75-positive and proliferating P75-positive prior to direct reprogramming (shown in **A**). *N* = 7 wells from 3 embryos; two-tailed unpaired Student's t-test, ***p* = 0.0028. (**C**) Representative immunofluorescent micrographs of p3 skin cells derived from e14.5 Wnt1-Cre; tdTomato mice. Cells were transduced with BAM factors and GFP and cultured for 2 weeks without doxycycline (no reprogramming induction). (**D**) Quantification of the percentage of total cells that are GFP-positive in the NC and non-NC lineage after 2 weeks in culture without doxycycline (shown in **C**). *N* = 6 wells from 3 embryos; two-tailed unpaired Student's t-test, *p* = 0.1411. (**E**) Quantification of the percentage of total cells that are NC lineage after two weeks with or without reprogramming induction. No dox, *n* = 6 wells; Dox, *n* = 7 wells; from 3 embryos; two-tailed unpaired Student's t-test, ****p* = 0.0003. (**F**) Quantification of the percentage of total cells that are iNs in the NC and non-NC lineage after 2w in culture

without doxycycline (shown in **C**). *N* = 6 wells from 3 embryos; two-tailed unpaired Student's t-test, $^{**}p = 0.0017$. (**G**) Immunofluorescent micrographs of p3 skin cells derived from e14.5 C57BL/6J mice after 3 weeks of direct iN reprogramming. Images are representative of types of neurons observed from three independent biological replicates (embryos). Yellow arrows indicate characteristic punctate staining of VGLUT1 along the neurite. **A** and **C** micrograph scale bar is 100 μm and **G** scale bar is 50 μm. Error bars represent mean +/- s.e.m.

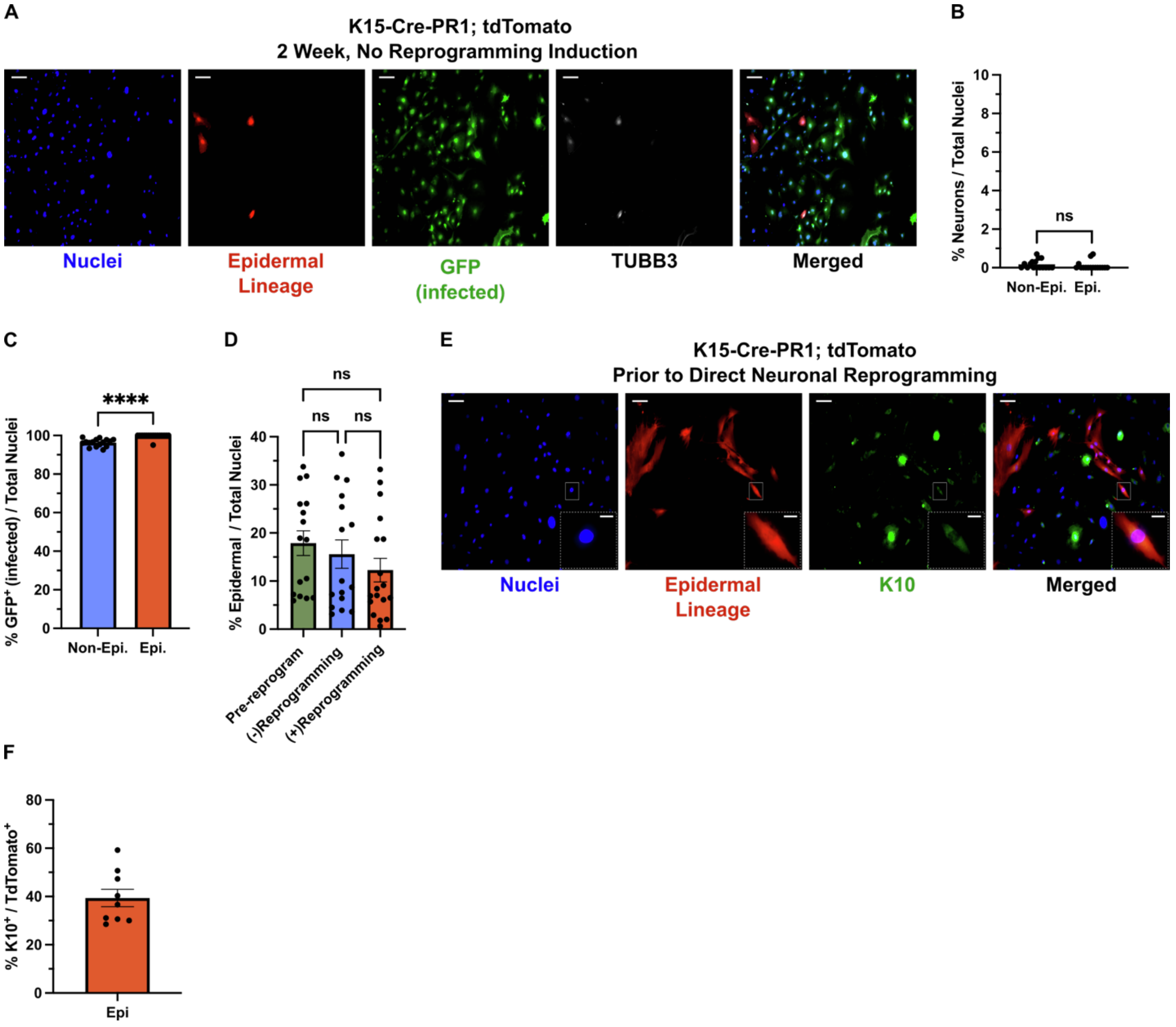

Figure S2 Transduction efficiency, baseline iNs, and direct reprogramming effect on epidermal cell survival. (**A**) Representative immunofluorescent micrographs p3 skin cells derived from e14.5 K15-Cre^{PR1}; tdTomato mice. Cells were transduced with BAM factors and GFP and cultured for 2w without doxycycline (no direct reprogramming induction). Cells shown were treated with mifepristone for three passages prior to direct reprogramming. (**B**) Percentage of total cells that are iNs after 2 weeks in culture without doxycycline (shown in **A**). Two-tailed unpaired Student's t-test, p = 0.3572. (**C**) Quantification of the percentage of total cells that are GFP-positive after 2 weeks in culture without doxycycline (shown in **A**). Two-tailed unpaired Student's t-test, ****p < 0.0001. **B** and **C,** N = 16 wells from 5 embryos. (**D**) Quantification of the percentage of total cells that are epidermal lineage. Pre-reprogram, n = 16 wells from 5 embryos; (-)Reprogramming, n = 18 wells from 5 embryos; (+)Reprogramming, n = 16 wells from 5 embryos; one-way ANOVA with Tukey's multiple comparisons test, p = 0.3226. (**E**) Representative immunofluorescent micrographs of p3 skin cells derived from e14.5 K15-Cre^{PR1}; tdTomato mice prior to direct iN reprogramming. Cells shown were treated with mifepristone for three passages prior to staining and imaging. (**F**) Quantification of the percentage of total tdTomato cells that are K10 positive. N = 9 wells from 2 embryos. For all micrographs main scale bar is 100 μm and any insert is 25 μm. Error bars represent mean +/- s.e.m.

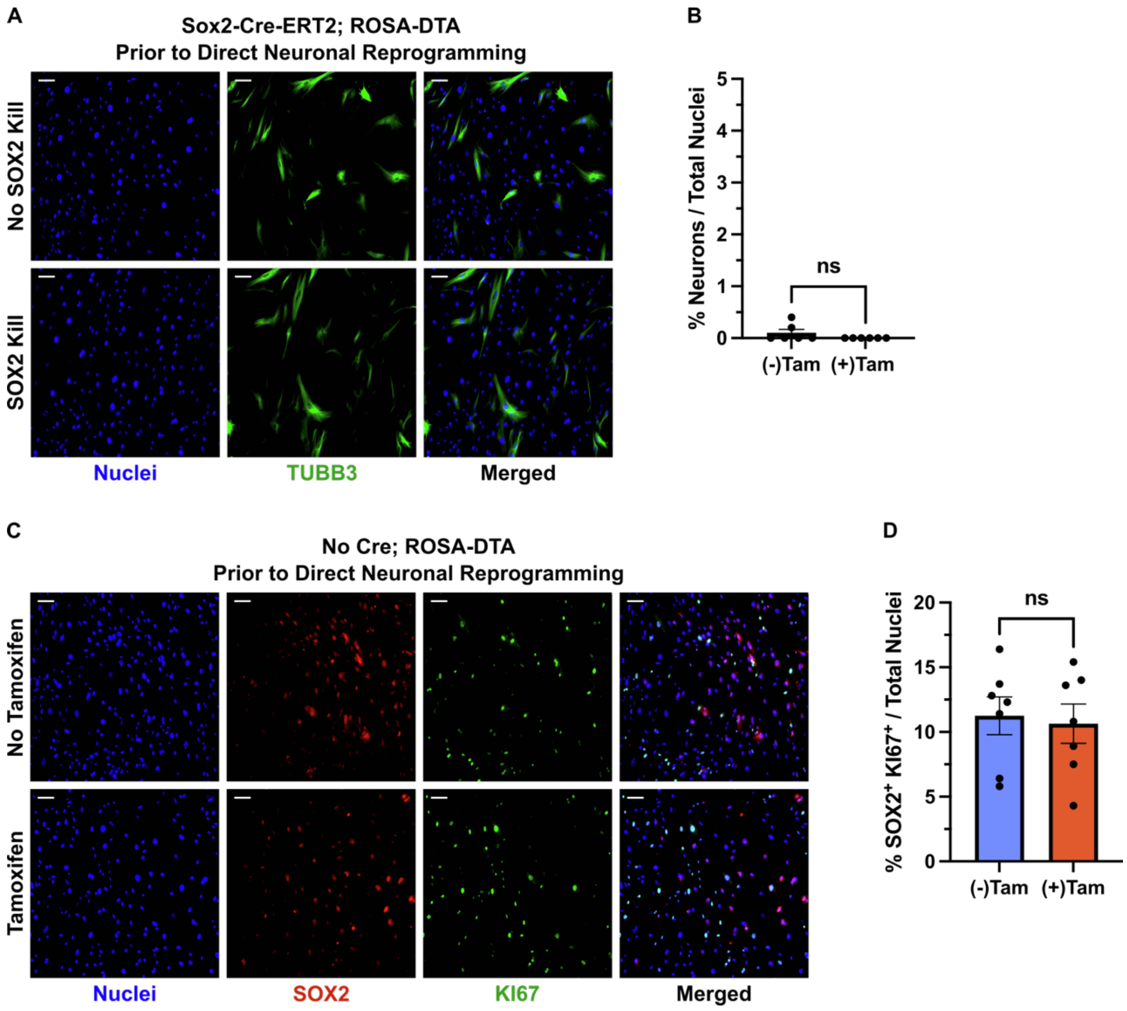

Figure S3 iNs in culture after NC precursor ablation prior to direct reprogramming and effect of tamoxifen alone on NC precursor number.
(**A**) Representative immunofluorescent micrographs of p3 skin cells derived from e14.5 Sox2-Cre^{ERT2}; ROSA-DTA mice prior to direct reprogramming. (**B**) Quantification of the percentage of total cells that are iNs prior to direct reprogramming with or without tamoxifen treatment (shown in **A**). (-)Tam, n = 6 wells; (+)Tam, n = 6 wells; from 2 embryos; two-tailed unpaired Student's t-test, p = 0.1739. (**C**) Representative immunofluorescent micrographs of p3 skin cells prior to direct reprogramming. Cells are derived from e14.5 Rosa-DTA mice (no Cre control). (**D**) Quantification of the percentage of total cells that are NC precursors as shown in **c**. (-)Tam, n = 7 wells; (+)Tam, n = 7 wells; from 2 embryos; two-tailed unpaired Student's t-test, p = 0.7748. For all micrographs scale bar is 100 μm. Error bars represent mean +/- s.e.m.

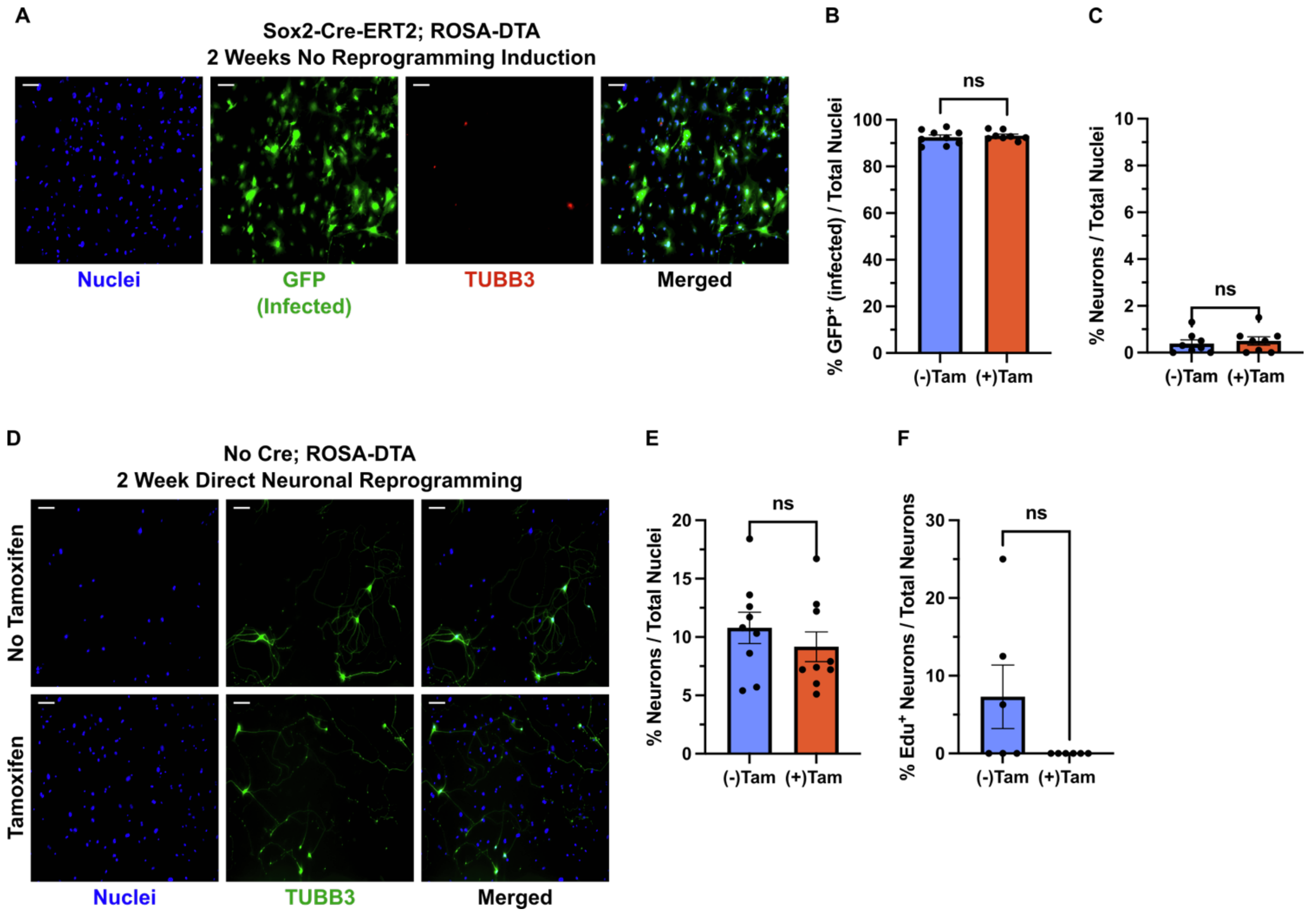

Figure S4 Transduction efficiency, baseline iNs without direct reprogramming induction, effect of tamoxifen alone on iNs, and EdU$^+$ iNs.

(**A**) Representative immunofluorescent micrographs of p3 skin cells derived from e14.5 Sox2-Cre^{ERT2}; ROSA-DTA mice. Cells were transduced with BAM factors and GFP and cultured for 2w without doxycycline (no direct reprogramming induction). (**B**) Quantification of the percentage of total cells that are GFP-positive after 2w in culture without doxycycline (shown in **A**). (-)Tam, n = 9 wells; (+)Tam, n = 8 wells; from 3 embryos; two-tailed unpaired Student's t-test, p = 0.6091. (**C**) Quantification of the percentage of total cells that are iNs after 2w in culture without doxycycline (shown in **A**). (-)Tam, n = 8 wells, (+)Tam, n = 8 wells; from 3 embryos; two-tailed unpaired Student's t-test, p= 0.6404. (**D**) Representative immunofluorescent micrographs of p3 skin cells after 2w of direct iN reprogramming. Cells are derived from e14.5 ROSA-DTA mice (no Cre control). (**E**) Quantification of the percentage of total cells that are neurons after 2w of reprogramming with or without tamoxifen treatment prior to direct reprogramming (shown in **D**). (-)Tam, n = 9 wells; (+)Tam, n = 9 wells; from 3 embryos; two-tailed unpaired Student's t-test, p = 0.3951 (**F**) Quantification of the percentage of total iNs that are EdU positive after 2w of direct reprogramming. From day 0 of direct reprogramming, cells were treated with 10 μM EdU every 2-3 days. (-)Tam, n = 6 wells; (+)Tam, n = 6 wells; from 2 embryos; two-tailed unpaired Student's t-test, p = 0.1044. For all micrographs scale bar is 100 μm. Error bars represent mean +/- s.e.m.

Supplemental Table

Table S1. Primers Used for Genotyping

Primer	Sequence (5’ => 3’)	Band Size
Cre-forward	AAAATTTGCCTGCATTACCG	553 bp
Cre-reverse	ATTCTCCCACCGTCAGTACG	553 bp
X-forward	GATCATGACCGCCGTAGG	310 bp
X-reverse	CATGAACTTGTCCCAGGCTT	310 bp
tdTomato-WT-forward	AAGGGAGCTGCAGTGGAGTA	297 bp
tdTomato-WT-reverse	CCGAAAATCTGTGGGAAGTC	297bp
tdTomato-mutant-forward	CTGTTCCTGTACGGCATGG	196 bp
tdTomato-mutant-reverse	GGCATTAAAGCAGCGTATCC	196 bp
DTA-WT	GGAGCGGGAGAAATGGATATG	650 bp
DTA-mutant	GCGAAGAGTTTGTCCTCAACC	340 bp
DTA-common	AAAGTCGCTCTGAGTTGTTAT	N/A

Supplemental Experimental Procedures

Genotyping

Using mouse ear notches, DNA extraction and PCR was performed using the RED-Extract-N-Amp PCR kit (Sigma). All Cre mice used a universal Cre primer set with hemoglobin X primers as an internal positive control. The following thermal cycler settings were used for the Cre + X reaction: 1x(94.0°C for 3 min), 35x(94.0°C for 1 min, 58°C for 30 sec, 72°C for 1.5 min), 1x(72°C for 10 min). For all other genotyping reactions thermal cycler settings were as listed on their respective Jackson strain page website.

Viral production

Viral plasmids and preparation were done as previously described (Vierbuchen et al., 2010). The following third generation lentiviral packaging plasmids were used: pMD2.G (addgene, #12259), pRSV-Rev (addgene, #12253), and pMDLg/pRRE (addgene, #12251). For direct reprogramming, individual viral preparations were made for each of the following plasmids: Tet-O-FUW-Brn2 (addgene, #27151), Tet-O-FUW-Ascl1 (addgene, #27150), Tet-O-FUW-Myt1l (addgene, #27152), FUW-M2rtTA (addgene, #20342), and pLJM1-EGFP (addgene, #19319). HEK293T cells were maintained in feeder media in 10cm dishes and passaged at least once at 70-90% confluency (using 1X TrypLE, Thermo, 12605010) before transfection. Fresh media was exchanged, and cells were transfected at 70-90% confluency in the evening using the lipofectamine 2000 (Thermo, 11668019) manufacturer's protocol. Each 10 cm dish received the following concentration of plasmids: 2.5 μg pMD2.G, 2.5 μg pRSV-Rev, 5ug pMDLg/pRRE, 10 μg reprogramming plasmid. The following morning (~16-18 hours) media was exchanged and then 24 hours after that media containing virus was collected. Viral supernatant was spun down (5 min, 0.4 rcf) to remove cellular debris and then run through a 0.45 μm PVDF syringe filter (Sigma, SLHV033RS). Aliquots of filtered viral supernatant were stored at -80°C.

Fluorescence-activated cell sorting

P3 skin cells were sorted for tdTomato using a FACSAria machine (BD Biosciences) and analysis was performed using FACSDiva (BD Biosciences). Sorted positive and negative cells were then cultured and reprogrammed as described in "Direct neuronal reprogramming".

in vivo NC precursor cell ablation

Sox2-Cre^{ERT2}; Rosa-DTA mice at postnatal day 1 were treated with tamoxifen cream on their skin. To prepare the tamoxifen cream, 100 μL of 200 mg/mL (Z)-4-hydroxytamoxifen was dissolved in 20g of hand cream (Neutrogena) (Vasioukhin et al., 1999). This was then applied to the head and neck skin once a day for 5 consecutive days. The treated regions of the skin were then dissected and cultured as described in "Primary cell isolation and culture".

Immunocytochemistry

Unless indicated, the following steps all occur at room temperature. Fixation was performed with 4% PFA for 15 min. After fixation cells were washed 3X (5 min each) with PBS (Thermo, 14190144) and stored at 4°C until staining. Cells were permeabilized with 0.3% Triton X-100 (Sigma, X100-100ML) in PBS for 10 min and then washed with PBS three times (5 min each). Next, 10% normal goat serum (NGS, Jackson ImmunoResearch, 005-000-121) in PBS was applied for 1 hour. The following primary antibodies in 1% NGS were incubated in specified dilutions overnight at 4°C: 1:500 mouse BIII tubulin (Abcam, ab78078), 1:100 rabbit SOX2 (Millipore, AB5603), 1:200 mouse KI67 (Abcam, ab279653), 1:1000 chicken GFP (Aveslabs, GFP-1010), 1:500 rabbit RFP (Rockland, 600-401-379), 1:500 rabbit VGLUT1 (Abcam, ab272913), 1:250 rabbit TBR1 (Abcam, ab183032), 1:500 rabbit GABA (Thermo, PA5-32241), 1:100 rabbit P75 (Abcam, ab52987), and 1:100 mouse K10 (Novus, NBP2-61736) . After overnight incubation, cells were washed 3X with PBS (5 min, 10 min, 10 min) and then incubated with secondary antibodies in 1% NGS for 1 hour. The following Alexa Fluor antibodies were used at a dilution of 1:400: goat anti-chicken 488 (Thermo, A-11039), goat anti-mouse 488 (Thermo, A-11001), goat anti-rabbit 488 (Thermo, A-11008) goat anti-mouse 568 (Thermo, A-11004), goat anti-rabbit 568 (Thermo, A-11011), goat anti-mouse 647 (Thermo, A3728), and goat anti-rabbit 647 (Thermo, A32733). After secondary antibodies, cells were washed 3X with PBS (5 min, 10 min, 10 min). 1:1000 Hoechst 33342 in PBS was applied for 10 min followed by 3X PBS wash (5 min, 10 min, 10 min). Fully stained cells were stored in PBS at 4°C in the dark. Each experimental condition and combination of antibodies include a secondary antibody only negative control to determine background levels of fluorescence. Primary antibodies were also tested for specificity in primary tissue of known expression.

Microscopy and image quantification
Fluorescent cells were imaged using a Zeiss AxioObserver D1 inverted microscope with Zeiss AxioVision software. Cells from each embryo were kept separate throughout the course of the experiments. Each well was split into 4 approximately equal imaging quadrants, and 1-2 images were taken per quadrant (4-6 images total). Cells tended to cluster together after reprogramming (Vierbuchen et al., 2010), and so this was the general area of focus in each quadrant to capture data from as many cells as possible. A cell type was determined by the presence or absence of a fluorescent maker, and not the level of fluorescence. For quantifying iNs, a cell must have both expression of BIII tubulin (TUBB3) and a small round soma with at least one process 3 times the length of the soma diameter. Using AxioVision, if brightness or contrast was amplified, it was done so uniformly across the entire image and in negative control images to ensure no false positives or negatives were being created. Images were exported as TIFF files and analyzed using ImageJ software. The ImageJ SNT plugin (Arshadi et al., 2021) was used for neuron morphology analysis (neurite length and complexity (Pillai et al., 2012)). Semi-automated neuron tracing was done using the A* search algorithm and cursor auto-snapping features of SNT. As the neurons were being visualized using a BIII Tubulin antibody with a strong signal that will stain the entire cell, the A* search algorithm worked well to guide neurite tracing. This tracing can be described as semi-automated because the user must manually click along the suggested neurite paths and always visually confirm that the suggested path is correct. Branch points off a primary neurite were always manually selected. If any errors were present, then fully manual tracing was done for the incorrect portion of the neurite path.

Electrophysiology
Conventional whole-cell patch-clamp recordings were conducted at the room temperature (22 ^{o}C) using Wnt1-Cre; tdTomato cells after 2 weeks of direct iN reprogramming. All recorded cells were tdTomato-positive (NC lineage). The cells, cultured on Geltrex coated coverslips, were transferred to 35-mm petri dishes for recordings, which were performed using an Axopatch-1D amplifier (Molecular Devices, USA) and an Axon Digidata 1440A acquisition system (Molecular Devices, USA). Recording electrodes (3 to 6 MΩ) were constructed from micropipette glass (World Precision Instruments, Inc., USA) using a P-87 pipette puller (Sutter Instrument Co., USA). The electrodes were filled with the intracellular solution composed of (in mM, pH 7.20 adjusted with KOH): 144 K^+-gluconate, 10 HEPES, 10 KCl, 2 EGTA, and 2 Mg-ATP. The extracellular solution consisted of (in mM, pH 7.35 adjusted with NaOH): 140 NaCl, 1 $MgCl_2$, 5.4 KCl, 15 HEPES, 2 $CaCl_2$ and 10 glucose. Action potentials, in current-clamp, were triggered by injecting a series of current steps from -20 pA to +200 pA (in 20-pA increments) for 1 s from the membrane potential of around -75 mV. A liquid junction potential of 16 mV was corrected under current-clamp condition with K^+-gluconate based internal solution. Voltage-gated ion currents, in voltage-clamp, were evoked by stepping the holding membrane potentials of -70 mV to a series of potentials from –80 mV to +60 mV (in 10-mV increments) for 400 ms. All electrical signals, acquired online using Clampex 10.7 software (Molecular Devices, USA), were digitized at 10 kHz and filtered at 2 kHz. The recording analysis were performed offline using Clampfit 10.7 software (Molecular Devices, USA).

References
Arshadi, C., Günther, U., Eddison, M., Harrington, K.I.S., and Ferreira, T.A. (2021). SNT: a unifying toolbox for quantification of neuronal anatomy. Nat Methods *18*, 374–377. https://doi.org/10.1038/s41592-021-01105-7.
Pillai, A.G., de Jong, D., Kanatsou, S., Krugers, H., Knapman, A., Heinzmann, J.-M., Holsboer, F., Landgraf, R., Joëls, M., and Touma, C. (2012). Dendritic Morphology of Hippocampal and Amygdalar Neurons in Adolescent Mice Is Resilient to Genetic Differences in Stress Reactivity. PLoS One *7*, e38971. https://doi.org/10.1371/journal.pone.0038971.
Vasioukhin, V., Degenstein, L., Wise, B., and Fuchs, E. (1999). The magical touch: Genome targeting in epidermal stem cells induced by tamoxifen application to mouse skin. Proceedings of the National Academy of Sciences *96*, 8551–8556. https://doi.org/10.1073/pnas.96.15.8551.
Vierbuchen, T., Ostermeier, A., Pang, Z.P., Kokubu, Y., Südhof, T.C., and Wernig, M. (2010). Direct conversion of fibroblasts to functional neurons by defined factors. Nature *463*. https://doi.org/10.1038/nature08797.
